# Zinc oxide and silver nanoparticles toxicity in the baker's yeast, *Saccharomyces cerevisiae*

**DOI:** 10.1371/journal.pone.0193111

**Published:** 2018-03-19

**Authors:** Imelda Galván Márquez, Mergan Ghiyasvand, Andrey Massarsky, Mohan Babu, Bahram Samanfar, Katayoun Omidi, Thomas W. Moon, Myron L. Smith, Ashkan Golshani

**Affiliations:** 1 Department of Biology and Ottawa Institute of Systems Biology, Carleton University, Ottawa, Ontario, Canada; 2 Department of Biology, Centre for Advanced Research in Environmental Genomics and the Collaborative Program in Chemical and Environmental Toxicology, University of Ottawa, Ottawa, Ontario, Canada; 3 Department of Biochemistry, University of Regina, Regina, Saskatchewan, Canada; 4 Agriculture and Agri-Food Canada, Ottawa Research and Development Centre (ORDC), Ottawa, Ontario, Canada; Institute of Materials Science, GERMANY

## Abstract

Engineered nanomaterials (ENMs) are increasingly incorporated into a variety of commercial applications and consumer products; however, ENMs may possess cytotoxic properties due to their small size. This study assessed the effects of two commonly used ENMs, zinc oxide nanoparticles (ZnONPs) and silver nanoparticles (AgNPs), in the model eukaryote *Saccharomyces cerevisiae*. A collection of ≈4600 *S*. *cerevisiae* deletion mutant strains was used to deduce the genes, whose absence makes *S*. *cerevisiae* more prone to the cytotoxic effects of ZnONPs or AgNPs. We demonstrate that *S*. *cerevisiae* strains that lack genes involved in transmembrane and membrane transport, cellular ion homeostasis, and cell wall organization or biogenesis exhibited the highest sensitivity to ZnONPs. In contrast, strains that lack genes involved in transcription and RNA processing, cellular respiration, and endocytosis and vesicular transport exhibited the highest sensitivity to AgNPs. Secondary assays confirmed that ZnONPs affected cell wall function and integrity, whereas AgNPs exposure decreased transcription, reduced endocytosis, and led to a dysfunctional electron transport system. This study supports the use of *S*. *cerevisiae* Gene Deletion Array as an effective high-throughput technique to determine cellular targets of ENM toxicity.

## Introduction

Engineered nanomaterials (ENMs) are increasingly integrated into everyday life. ENMs possess unique physical, chemical, and structural properties attributable to their small size (≤100 nm in at least one dimension) [1]. Zinc oxide nanoparticles (ZnONPs) and silver nanoparticles (AgNPs) are among the most commonly used ENMs. ZnONPs are present in numerous consumer products, especially in ultraviolet (UV) blocking cosmetics [2, 3]. ZnONPs effectively absorb UV-A and UV-B light through a process called band-gap absorption, and are less photoactive than titanium dioxide nanoparticles (TiO_2_NPs), which are also used in sunscreens [4]. The photocatalytic properties of ZnONPs have also been extensively studied in relation to degradation of organic pollutants, including various dyes and acetaldehyde that are used industrially and released as effluents [5–7]. ZnONPs also have biocidal/antimicrobial properties [8, 9] that are thought to be mediated by the release of zinc ions (Zn^2+^) and generation of reactive oxygen species (ROS) [10]. These properties have been extensively studied in relation to potential antiviral and anti-cancer treatments [11–15], as well as antifouling agents [16].

On the other hand, AgNPs are well known as effective growth inhibitors of a wide range of Gram-positive and Gram-negative bacteria and some viruses, and as such they are incorporated into a wide range of consumer products [17]. The antimicrobial actions of AgNPs may involve (i) release of silver ions (Ag^+^), which mainly bind to thiol-containing compounds thereby disrupting important cellular functions, including DNA replication, (ii) attachment to cell membranes, which disrupts the membrane potential, and (iii) generation of ROS [18]. The ability of AgNPs to generate ROS is applicable not only in antimicrobial/antiviral applications, but also in pollutant degradation [19–20], as well as cancer treatment [20–21].

Despite the potentially beneficial properties of ENMs, concerns about their safety have been raised over the past decade. It has been estimated that sludge-treated soil would be the main environmental compartment for deposition of ZnONPs and AgNPs, and could accumulate 1.6–23.1 and 0.5–4.1 μg/kg/y of NPs, respectively [22]. This suggests that organisms in the soil would be at a greater risk to adverse effects of ENMs. The overall health of microorganism populations is of particular concern given their key contributions to the ecosystem, including nitrogen fixation and nutrient cycling. Several studies have shown a plethora of effects by ZnONPs and AgNPs on various bacterial species, including oxidative stress and damage, as well as uptake and damage to various cellular components (reviewed by [23]). However, very few studies have addressed the toxicity of ZnONPs and AgNPs in eukaryotic microorganisms. Kasemets et al. [24] examined the toxicity of ZnONPs in the budding yeast (*Saccharomyces cerevisiae*) and reported that growth was inhibited by 80% at 250 mg/L ZnO for both nano-scale and bulk forms. Moreover, it was suggested that growth inhibition was due to the release of Zn^2+^ ions and possible induction of oxidative stress. In contrast, a lower concentration of 50 mg/L AgNPs was necessary to inhibit yeast growth [25]. The latter study reported that cellular proteins, amino acids, and RNA molecules, as well as the plasma membrane were possibly affected by AgNPs. Notably, generation of hydroxyl radicals and induction of apoptosis were suggested as toxicity mechanisms for AgNPs in another yeast species, *Candida albicans* [26].

The current study uses a Gene Deletion Array (GDA) as a platform for a high-throughput functional genomic screening to enhance our understanding of ENM toxicity. The GDA is comprised of ≈4600 non-essential gene deletion strains of *S*. *cerevisiae*. We predict that strains with deletion of genes in a parallel, redundant pathway to that targeted by ZnONPs and/or AgNPs, will have increased sensitivity to these NPs, as was demonstrated for other compounds [27–30]. Highly sensitive strains are then categorized according to the cellular activity and function of the deleted genes, in order to deduce cellular pathways that are affected by these NPs. Thus, we examined the chemical-genetic profiles of ZnONPs and AgNPs using the GDA platform and validated the findings with follow-up assays.

## Materials and methods

### 2.1. Chemicals

ZnONPs in powder form (catalog #544906; particle size 50–70 nm) were purchased from Sigma-Aldrich (Oakville, ON, Canada). An aqueous dispersion of AgNPs (2 mg/mL; 31% silver content; particle size 1–10 nm) was purchased from Sciventions Inc (Toronto, ON, Canada). MTT (3-(4,5-dimethylthiazol-2-yl)-2,5-diphenyltetrazolium bromide), sodium azide, glucose, MES (2-morpholinoethanesulfonic acid), DMSO (dimethyl sulfoxide), geneticin (G418), CCCP (carbonyl cyanide 3-chlorophenylhydrazone), and MUG (4-methylumbelliferyl-D-galactopyranoside) were purchased from Sigma-Aldrich. DiSBAC_2_(3) [bis-(1,3-diethylthiobarbituric acid)trimethine oxonol] was purchased from Life Technologies (Carlsbad, CA, USA).

### 2.2. NPs preparation and characterization

ZnONPs were suspended in 95% ethanol at a concentration of 10 mg/mL and sonicated (Vibra Cell VCX130, Sonics & Materials Inc., Newtown, CT, USA) for 5 min. This stock solution was then diluted to 1 mg/mL in YPD medium (1% yeast extract, 2% peptone, 2% dextrose). The aqueous solution of silver nanoparticles (AgNPs; 0.62 mg/mL total silver) was diluted to 0.095 mg/mL in YPD medium.

The size of ZnONPs and AgNPs was assessed using dynamic light scattering (DLS) with a Zetasizer NanoZS according to the manufacturer’s guidelines (Malvern Instruments Ltd. Malvern, Worcestershire, UK). Each sample was measured at least 3 times, and the obtained values were used to calculate the average. The size of nanoparticles was verified using transmission electron microscopy (TEM). The TEM images of the nanoparticles were obtained using a FEI Tecnai G2 Spirit TEM with a Lab6 emitter operating at 120 kV. Prior to analysis, nanoparticles were prepared as follows. The ZnONPs powder was dispersed in ethanol (EtOH) to form a ZnO/EtOH dispersion. The aqueous dispersion of silver nanoparticles was diluted in water. Both ZnO/EtOH and Ag/H_2_O dispersions were sonicated for 10 min using a BRANSON 3510 Ultrasonic Cleaner (Marshall Scientific, Hampton, NH, USA). The TEM specimen was prepared by placing a small drop of ZnO/EtOH or Ag/H2O dispersion onto a TEM copper grid supported with carbon film, then dried at room temperature.

### 2.3. Yeast strains and growth conditions

Yeast cells (*S*. *cerevisiae*) strains S288C (*MATα SUC2 gal2 mal2 mel flo1 flo8-1 hap1 ho bio1 bio6*) or W303 (*MAT*a*/MATα {leu2-3*,*112 trp1-1 can1-100 ura3-1 ade2-1 his3-11*,*15}*) were grown in YPD medium at 30 °C for 1–2 days. Deletion strains were arrayed on YPD medium that was supplemented with 2% agar and 200 μg/mL kanamycin (G418).

### 2.4. Minimum inhibitory concentrations and sensitivity analyses

The minimum inhibitory concentrations (MIC_100_) for ZnONPs and AgNPs were assessed according to the Reference Method for Broth Dilution Antifungal Susceptibility Testing of Yeasts M-27-A2 [31] and drop-out assays [32]. An overnight culture of yeast (S288C) cells was 10-fold serially diluted to obtain 10^5^ cells/mL. For microdilution assays, different concentrations of ZnONPs (0.25–5 mg/mL) and AgNPs (0.005–0.1 mg/mL) were assayed to determine the corresponding MIC values. Microtitre plates were incubated at 30 °C for 24 h, and inhibitory activity was evaluated by reading the absorbance at 600 nm using a FLUOstar Optima multi-mode microplate reader (BMG Labtech, Ortenberg, Hessen, Germany). The MIC_100_ (the lowest concentration that resulted in complete inhibition of visible growth in liquid medium) at 48 h was calculated using the equation:

$$100-[(Abs_{exp}-Abs_{blank})÷(Abs_{carrier}-Abs_{carrier\;blank})]\times 100$$ (1)

Where Abs_exp_ is the absorbance of the treated (with ZnONPs or AgNPs) sample (medium and yeast cells), Abs_blank_ is the absorbance of the medium (no cells and no nanoparticles), Abs_carrier_ is the absorbance of the carrier (solvent used to prepare the nanoparticles) with yeast cells used as a growth control, and Abs_carrier blank_ is the absorbance of the medium and the carrier without cells. The MIC values were used as a guide to determine the sub-inhibitory concentrations of ZnONPs and AgNPs for GDA analyses. Colonies from two plates were randomly chosen from the GDA mutant set and replicated onto YPD-agar plates containing a range of ZnONPs (0.1–1.5 mg/mL) or AgNPs (0.003–0.12 mg/mL) concentrations. Plates were incubated at 30 °C for 1–2 days and colony sizes were measured to identify the concentration of ZnONPs or AgNPs that reduced the colony size by at least 30% in 5–10% of the strains compared to control. The appropriate sub-inhibitory concentrations determined in this way were used for full scale GDA analyses.

### 2.5. High-throughput phenotypic screening

Approximately 4600 haploid gene deletion strains of *S*. *cerevisiae* were exposed to a sub-inhibitory concentration of ZnONPs and AgNPs nanoparticles (1 mg/mL and 0.095 mg/mL, respectively). Plates were incubated for 1–2 d at 30 °C and digital images of these plates were acquired. The area of the colonies was determined from the images as explained elsewhere [33]. The size of each colony was compared to the average size for all colonies on both experimental and control plates [29, 34]. Each experiment was carried out in triplicate. Colonies with size reduction of at least 50% in at least two replicate experiments were classified as highly sensitive strains. Functional clustering of the highly sensitive mutants was performed according to eukaryotic orthologous groups (KOG), GeneMania, and Gene Ontology (GO) term finder through the Saccharomyces Genome Database (SGD) [35–37].

Mutant strains identified as highly susceptible to ZnONPs and/or AgNPs by the large-scale screening above were verified using drop-out assays [32, 38] on YPD-agar plates with and without the sub-inhibitory concentration of 1 mg/mL ZnONPs or 0.095 mg/ml AgNPs. Plates were incubated at 30 °C for 1–2 d and enumeration of colony forming units was performed to estimate growth inhibition.

### 2.6. Cell membrane disruption analyses

#### 2.6.1. Liposomes

Membrane disruption by ZnONPs was examined according to Cruz et al. [39]. Briefly, liposomes were prepared as described by Cheetham et al. [40] with dioleoylphosphatidylcholine to encapsulate carboxyfluorescein in large unilamellar vesicles (LUVs; ~100 nm in diameter). LUVs were suspended in iso-osmotic buffer (100 mM NaCl, 10 mM HEPES, pH 7.4) and aliquoted into a 96-well microplate (black/clear Optilux flat bottom; BD Bioscience, San Jose, CA, USA). ZnONPs were diluted in the iso-osmotic buffer and added to the LUVs at a final concentration range of 0.03 to 0.2 mg/mL. The iso-osmotic buffer was used as a negative control. Fluorescence threshold value was identified by the fluorescent signal of the LUV suspension alone. Fluorescence intensity (485 nm ex./528 nm em.) was measured using a Cytation 5 cell imaging multi-mode reader (BioTek, Winooski, VT, USA). Liposomes were incubated for 1 h at room temperature in the dark prior to obtaining fluorescent emission readings. The percent leakage (%L) was calculated by the equation:

$$\%L=\left\lfloor \left(F-F_{0}\right)÷\left(F_{100}-F_{0}\right)\right\rfloor \times 100$$ (2)

Where F is fluorescence intensity after incubation of liposomes with ZnONPs or iso-osmotic buffer (negative control), F_0_ is the fluorescence intensity of the liposomes in the buffer solution, F_100_ is fluorescence intensity corresponding to 100% leakage after the addition of triton X-100 (10% v/v) for 10 min.

#### 2.6.2. Trypan blue stain

Membrane integrity was examined using the vital dye trypan blue [41, 42]. Yeast cells were grown overnight in YPD medium to mid-log phase and adjusted to approximately 10^4^ cells/mL based on absorbance at 600 nm. Yeast cultures were subjected to concentrations of ZnONPs ranging from 0.1 to 1.5 mg/mL. Control cells were treated with equivalent volumes of 95% ethanol. Overnight cultures of treated cells were adjusted to a density of 10^7^ cells/mL, and mixed (1:1) with a 0.4% trypan blue solution. The viable (unstained) and non-viable (stained) cells were counted separately using a hemocytometer and a microscope (CARL ZEISS #4649608, Oberkochen, Ostalbkreis, Germany). Observations were performed in triplicate and averaged.

#### 2.6.3. Depolarization analysis

The effect of ZnONPs on cell membrane depolarization was examined using flow cytometry. Yeast cells were incubated overnight and cell density was adjusted to 10^7^ cells/mL. Yeast cultures were treated for 3 h with either 1.0 mg/mL ZnONPs, 10 mM citric acid (negative control), or 20 μM CCCP (positive control). Cells were then centrifuged (9300 *x g*, 2 min) and washed twice with phosphate buffered saline (PBS). After staining with 5 μM DiSBAC_2_(3) for 30 min at 23 °C, the cells were subjected to flow cytometry analysis (BD Accuri C6, BD Biosciences, East Rutherford, NJ, USA) using a red laser (488 nm ex./585±40 nm em.). Samples were injected at a speed of 36 μL/min, and 10–10000 events were measured per sample. Forward scatter (FSC) and side scatter (SSC) were simultaneously measured. An increase in fluorescence intensity in the FL2-H range of 10^4^–10^5^ was expected for cells with depolarized membranes [43].

### 2.7. Cell wall disruption analysis

Cell wall disruption was assessed according to Cruz et al. [39]. Yeast cells were grown in YPD with sub-inhibitory concentrations of ZnONPs (0.5–1.5 mg/mL) overnight at 30 °C with constant shaking at 150 rpm. Cell density was adjusted to 10^7^ cells/mL and a 2 min sonication treatment was performed using a 3 mm microtip probe with amplitude set to 20%, a 15 s pulse and a 3 s interval between pulses (Vibra Cell VCX130, Sonics & Materials Inc., Newtown, CT, USA). Cell viability of sonicated and non-sonicated cells, with and without ZnONPs exposure, was measured by colony counts using drop-out assay analysis. Each experiment was performed in triplicate.

### 2.8. Transcription rate analysis

The effect of AgNPs on transcription was examined according to Vidal-Aroca et al. [44]. Briefly, yeast cells (strain W303) were transformed with the expression vector p416, which contains a galactose inducible β-galactosidase gene [45, 46]. Transformed cells were grown in a synthetic medium lacking uracil (SC-URA) supplemented with 2% glucose. Cells were harvested (0.3–0.6 OD) and washed twice before adding SC-URA medium containing 2% galactose. Cell density was adjusted to 10^7^ cells/mL and cultures were aliquoted into 96-well microtitre plates, where yeast cells were exposed to a range of sub-inhibitory concentrations of AgNPs (0.9–9.0 μg/mL). The transcription inhibitor 6-azauracil (48 μg/mL) was used as a positive control. Plates were incubated at 30 °C for 6 and 10 h. A 20 μL aliquot from each well was transferred into a 96-well microplate (black/clear Optilux flat bottom; BD Bioscience, San Jose, CA, USA) containing 80 μL Z-buffer (in M: 0.06 Na_2_HPO_4_•7H_2_O, 0.04 NaH_2_PO_4_•H_2_O, 0.01 KCl, 0.001 MgSO_4_, 0.05 β-mercaptoethanol, pH 7) and the absorbance at 600 nm was measured with a Cytation 5 cell imaging multi-mode plate reader (BioTek, Winooski, VT, USA). The reaction was initiated by adding 25 μL MUG (1 mg/mL in DMSO) to each well, followed by a 15 min incubation at room temperature. The reaction was stopped by adding 30 μL 1 M Na_2_CO_3_. β-Galactosidase activity was quantified by measuring fluorescence of the product MUB (390 nm ex./475 nm em.). MUB units for each replica (samples and controls) were calculated with the equation:

$$MUB=F_{390/475}÷\left(t\times Abs_{595}\right)$$ (3)

Where F_390/475_ is the sample fluorescence at the end of the reaction, t is the time of reaction in minutes (min), and Abs_595_ is the absorbance of the cell suspension.

### 2.9. Cellular respiration analysis

The MTT assay was used to assess cellular respiration as described elsewhere [47]. Briefly, yeast cells (S288C) were pelleted from overnight cell cultures, resuspended in distilled water, and incubated overnight at 30 °C in order to starve the cells. Cells were pelleted and resuspended in distilled water at a 1:2 ratio. A 15 μL aliquot of the cell suspension was added to a 1.5 mL microcentrifuge tube containing 100 μL of each 100 mM MES, 1 M glucose, and 5 mg/mL MTT. Sub-inhibitory concentrations of AgNPs (0.75–17.5 μg/mL) were added and volumes were adjusted to 1 mL using distilled water. Sodium azide (2.5 mM) was used as an electron transport chain inhibitor (positive control) and glucose (100 mM) was used as a negative control. Cells were incubated at 30 °C for 60 min, then placed on ice for 5 min and pelleted. The cells were resuspended in DMSO to dissolve the formazan salt. Samples were centrifuged and 100 μL of each supernatant was transferred to a microtitre plate. MTT reduction was determined using a FLUOstar plate reader (BMG Labtech; Ortenberg, Hessen, Germany) at 595 nm. Each experiment was repeated five times.

### 2.10. Fluid-phase endocytosis analysis

The lucifer yellow (LY) uptake assay was performed as described previously [48, 49] to estimate fluid-phase endocytosis of yeast cells. Briefly, yeast cells were grown to mid-log phase in YPD supplemented with 30 mg/L each of uracil, adenine, and tryptophan (YPD-UAT). Cell cultures were concentrated 10-fold by centrifugation. Aliquots (100 μL) of these concentrated cell suspensions were mixed with 100 μL treatment solution containing sub-inhibitory concentrations of AgNPs (40 or 80 μg/mL). A buffer (pH 7) containing 12.5 mM sodium phosphate, 2.5 mM sodium fluoride and 2.5 mM of the endocytosis inhibitor sodium azide (ATPase inhibitor), was used as a positive control. Samples were incubated at 30 °C for 15 min prior to the addition of LY for a final concentration of 4 mg/mL. Samples were then incubated at 30 °C for 1 h. Cells were pelleted and washed three times with 1 mL ice-cold succinate/azide buffer (50 mM succinic acid, 20 mM NaN_3_, pH 5.0). Pellets were resuspended in 200 μL of the buffer and observed by fluorescence microscopy (Axiophot, model, objective 40x and 100x, FITC optics; Zeiss, Germany). The percentage of fluorescent cells was calculated by analyzing at least six different fields of view, each with >20 cells.

### 2.11. Statistical analysis

The data are presented as mean ± standard error of the mean (SEM). Statistical analyses were conducted using SigmaPlot (SPW 12; Systat Software, Inc., San Jose, CA). A one-way Analysis of Variance (ANOVA) with a post-hoc Tukey method was used to assess significant differences in all assessed endpoints. A t-test was used to assess significant differences between control and positive control groups when applicable. In all cases p ≤ 0.05 was considered significant.

## Results and discussion

### 3.1. NPs characterization

**Fig 1:**
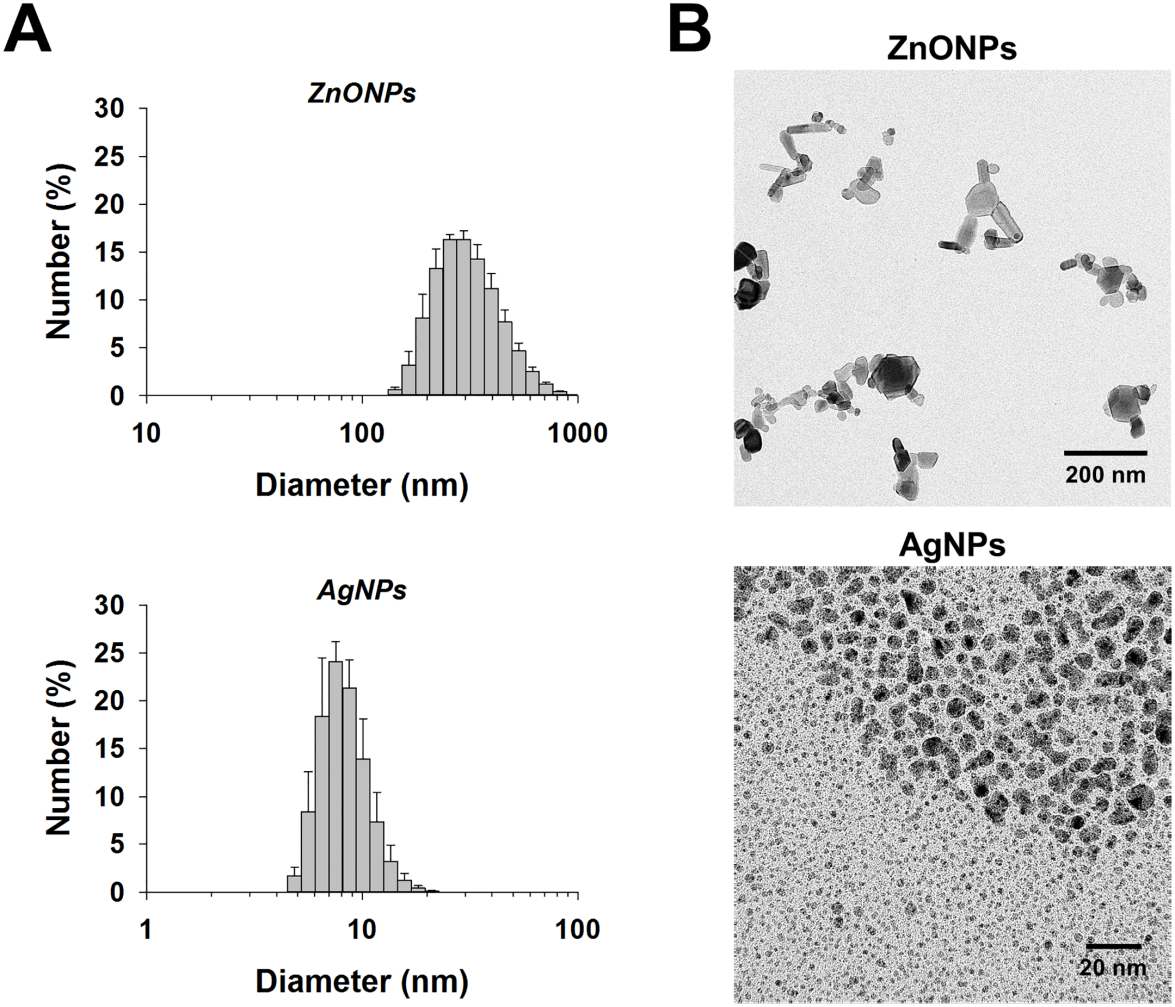
Size characterization of nanoparticles. (A) Dynamic light scattering (DLS) was used to assess the size of ZnONPs and AgNPs. Mean ± SEM are presented (n ≥ 3). (B) Transmission electron microscopy images of ZnONPs and AgNPs (Note: the scale bars are 200 and 20 nm for ZnONPs and AgNPs, respectively).

The average size of ZnONPs was 278 nm according to DLS measurements (Fig 1A). Subsequent TEM analysis showed agglomerates/aggregates of ~200 nm in size composed of individual particles that are between 20 and 70 nm in diameter (Fig 1B). The size of the individual ZnONPs is similar to the 50–70 nm size reported by the manufacturer. The average size of AgNPs was 9 nm according to DLS measurements (Fig 1A), which was confirmed by TEM analysis (Fig 1B). The measured size of AgNPs agrees with the 1–10 nm size range reported by the manufacturer.

It is noteworthy that the differences between ZnONPs and AgNPs potentially play a role in their differential toxicity potential. Specifically, ZnONPs appear to be less stable in solution as evidenced by the presence of agglomerates/aggregates. The aggregated ZnONPs may adhere to cell surfaces while individual ZnONPs may penetrate cell membranes to act internally. In contrast, AgNPs appear to be more stable in solution and of a rather uniform size, such that individual particles and/or Ag^+^ could potentially cross the cell membrane and affect internal structures/molecules. As far as the release of Ag^+^ is concerned, the AgNPs used in the current study were demonstrated previously to show minimal dissolution [50].

### 3.2. Minimum inhibitory concentrations and GDA analyses

The MIC_100_ values of ZnONPs and AgNPs using the yeast YPD-agar assay were 1.3<MIC_100_≤1.5 mg/mL and 0.10<MIC_100_≤0.12 mg/mL, respectively. On agar media, an ≈30% reduction in yeast colony size was observed at 1 mg/mL ZnONPs and 0.095 mg/mL AgNPs (note: these are nominal concentrations); these concentrations were therefore chosen for the high-throughput GDA phenotypic screenings. Out of the 4600 strains, 59 and 96 mutant strains were identified as highly sensitive to ZnONPs and AgNPs, respectively (S1 and S2 Tables). Drug sensitivity drop-out assays were used to confirm the sensitivities of selected mutant strains identified in the primary large scale screens. The results of these drop-out analyses confirmed the sensitivity of the *yjl095w*Δ, *ycl058c*Δ, and *yjl080c*Δ strains to ZnONPs and the sensitivity of the *yjr104c*Δ, *yn1037c*Δ and *ybr085w*Δ strains to AgNPs.

**Fig 2:**
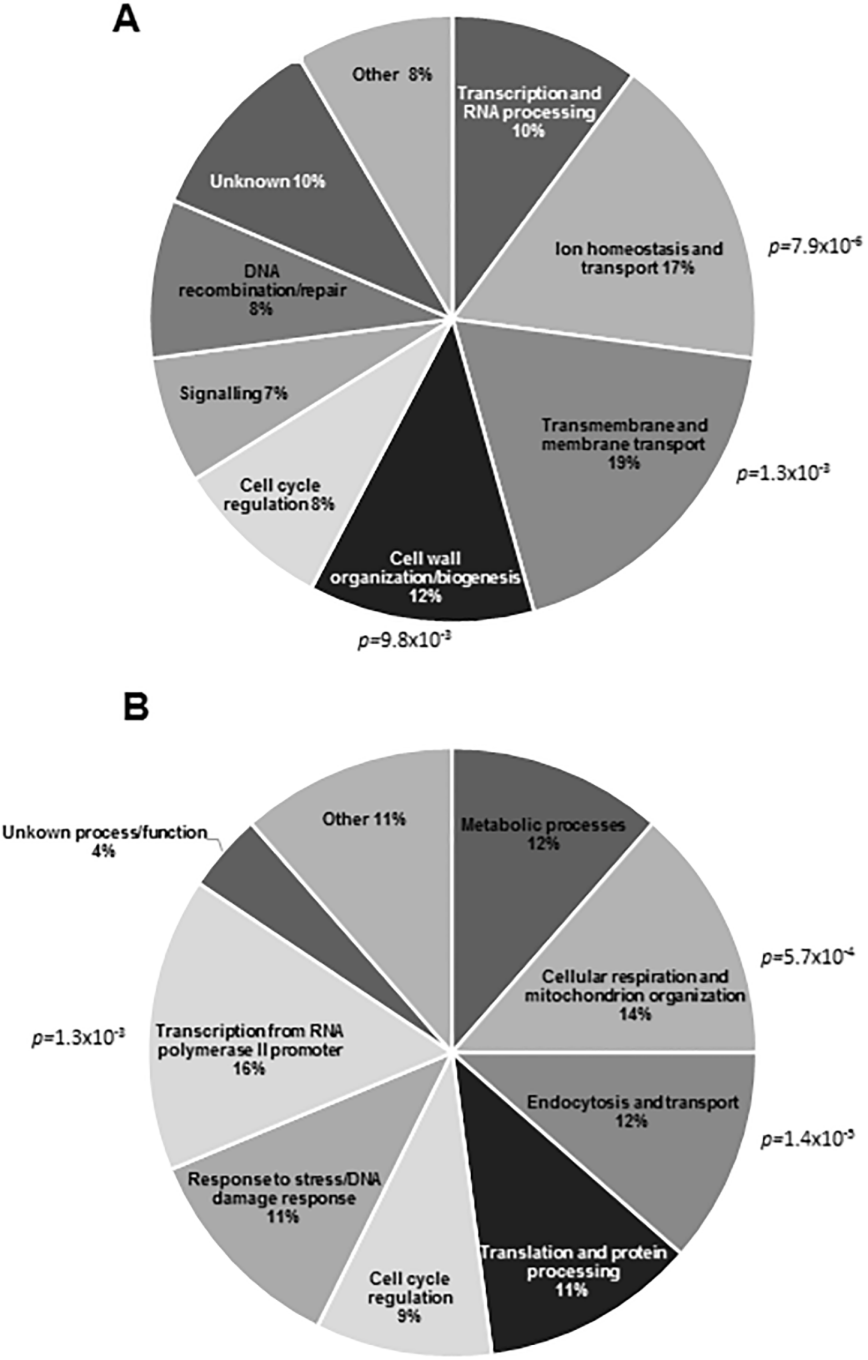
Functional distribution of deletion mutants that are highly sensitive to ZnONPs and AgNPs. (A) Clustering of the 59 most sensitive deletion mutant strains to 1 mg/mL ZnONPs reveal that mutants lacking genes involved in membrane and transmembrane transport, ion homeostasis and transport, and cell organization of biogenesis encompass the significantly enriched groups. (B) Clustering of the 96 most sensitive deletion mutant strains to 0.095 mg/mL AgNPs indicate that mutants lacking genes involved in transcription, cellular respiration, and endocytosis and vesicular transport represent the significantly enriched groups.

The highly sensitive mutants were clustered into functional categories (Fig 2). The highly sensitive ZnONPs mutant strains formed 3 functional categories with significant enrichment (Fig 2A). Transmembrane and membrane transport genes (*p-value ≤* 1.3x10^-3^) formed the most populated group (19%) of mutants highly sensitive to ZnONPs. Cellular ion homeostasis genes formed the second most populated group (*p-value ≤* 7.9x10^-6^), representing 17% of the most sensitive. Genes involved in cell wall organization/biogenesis formed another major group, representing 12% of the sensitive mutants (*p-value ≤* 9.8x10^-3^). Similarly, mutants sensitive to AgNPs could be categorized into several functional groups (Fig 2B). The largest groups were transcription and RNA processing (16%, *p-value ≤* 1.3x10^-3^), cellular respiration (14%, *p-value ≤* 5.7x10^-4^), and endocytosis and vesicular transport (12%, vesicle coat, *p-value ≤* 1.4x10^-5^). The above functional groups provide potential insights into the distinct modes of action of ZnONPs and AgNPs in yeast that were tested by secondary assays (see below).

### 3.3. Cell membrane disruption analyses in ZnONPs-treated yeast

GDA analysis identified a significant enrichment (19%, *p-value ≤* 1.3x10^-3^) of ZnONPs-sensitive strains with deletions of genes involved in transmembrane and membrane transport/organization. These genes include *PKR1*, which codes for a V-type proton-translocating ATPase assembly factor involved in transport of protons across intracellular membranes of organelles, and *ERG2* and *ERG28*, which code for proteins involved in biosynthesis of ergosterol, a sterol that has similar functions to cholesterol in animal cell membranes (e.g. membrane fluidity). Furthermore, there was an enrichment of mutants with deletions in genes involved in ion homeostasis (17%, *p-value ≤* 7.9x10^-6^), such as *FTR1* (codes for an ion transporter), *GEF1* (involved in cation homeostasis), and *SPF1* (mediates Ca^2+^ homeostasis). These results suggest that ZnONPs could disrupt intracellular processes, but most importantly impair the proper function of the cell membrane. Indeed, the ability of ZnONPs to disrupt the cell membrane in *E*. *coli* has been demonstrated previously and proposed as one of the antimicrobial mechanisms [51, 2].

**Fig 3:**
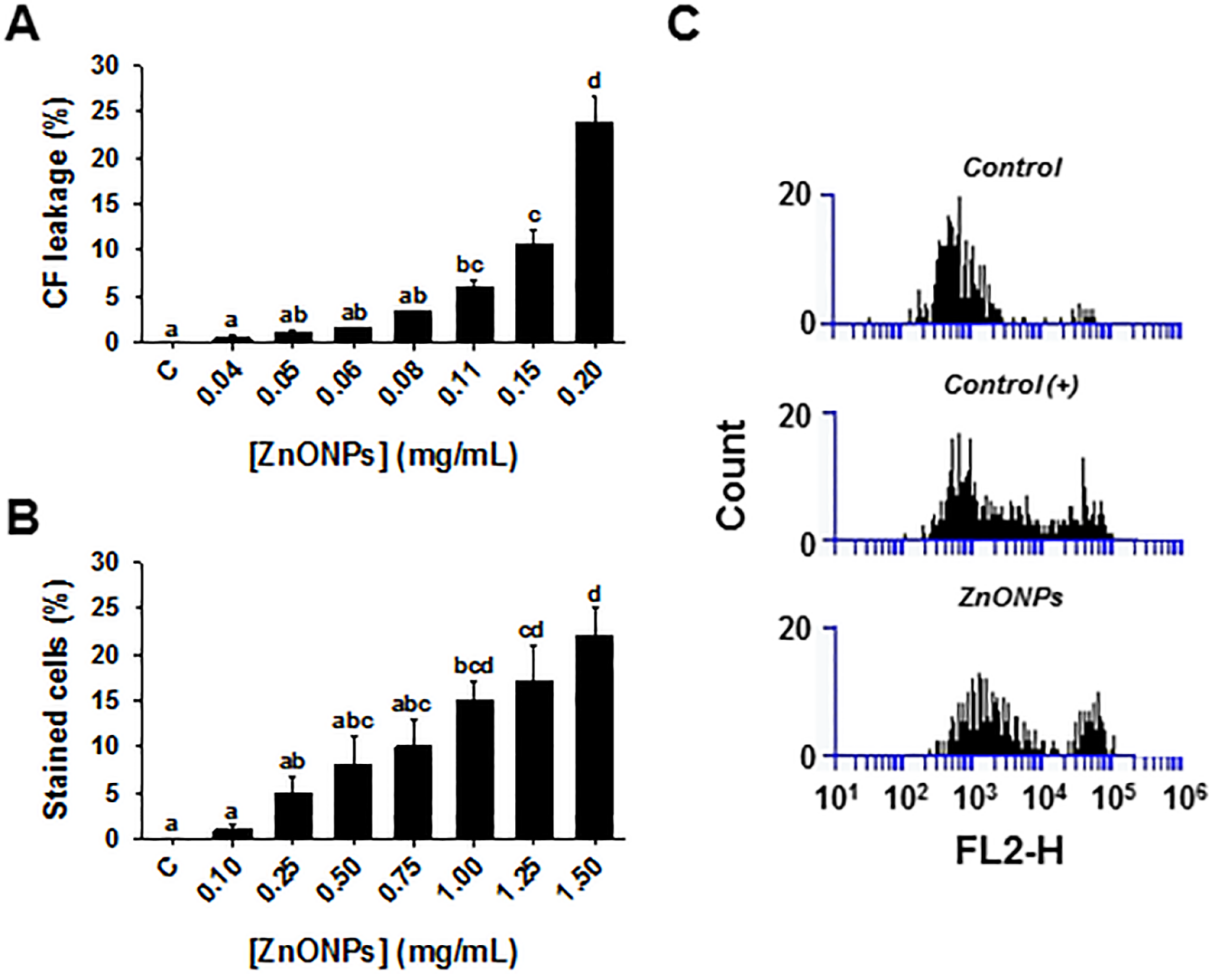
Cell membrane disruption by ZnONPs. (A) Carboxyfluorescein leakage from liposomes that were exposed to various concentrations of ZnONPs for 30 min. (B) Trypan blue exclusion assay in yeast cells following a 2 h exposure to various concentrations of ZnONPs. Mean ± SEM are presented (n ≥ 3). Significant differences are indicated with letters. (C) Membrane depolarization analysis in yeast cells that were subjected to: 0.01 mM citric acid (negative control), 20 μM CCCP (carbonyl cyanide 3-chlorophenylhydrazone; positive control), and 1 mg/mL ZnONPs. The histograms show the number (Count, Y-axis) of yeast cells in a sample with depolarized membranes (FL2-H interval = 10^4^–10^5^) and cells at resting potential (FL2-H = 10^2^–10^4^).

The effects of ZnONPs on the cell membrane were investigated using several approaches. There was a dose-dependent increase in fluorescence signal after treatment of carboxyfluorescein-containing liposomes with ZnONPs (0.03–0.2 mg/mL), such that treatment with the highest concentration resulted in 24% leakage (Fig 3A). The influence of ZnONPs on liposomes may be attributed to the release of Zn^2+^ ions that alter liposome conformation by an electrostatic interaction and modify permeability [52]. There was also a dose-dependent increase in the percentage of trypan blue stained cells observed upon treatment with ZnONPs (Fig 3B), which provides further evidence that ZnONPs disrupt the cell membrane resulting in either increased uptake or decreased efflux of trypan blue.

Moreover, treatment with 1.0 mg/mL ZnONPs affected cell membrane depolarization (Fig 3C). The assay relies on an electrical potential-sensitive fluorescent dye [DiSBAC_2_(3)] that only penetrates into cells with depolarized cell membranes and provides a characteristic fluorescent signal when it binds to intracellular proteins. The increase in fluorescent signal due to ZnONPs exposure was comparable to that of the proton ionophore CCCP (a depolarizing agent) used as a positive control. Both treatments showed a second peak in the fluorescent region (10^4^−10^5^) on the FL2-H axis, indicating that the dye was able to enter cells and bind to intracellular proteins.

The results of the three methods that assessed cell membrane integrity were consistent in indicating that ZnONPs disrupt the cell membrane of yeast. Altogether our observations validate the inference from the chemical-genetic profile analysis that yeast cell membrane and membrane transport are altered by ZnONPs, similar to observations with bacterial cells (e.g. *E*. *coli* [53]). By extension, ZnONPs could alter ion homeostasis, an effect that was previously reported in human cells [54].

### 3.4. Cell wall disruption analysis in ZnONPs-treated yeast

**Fig 4:**
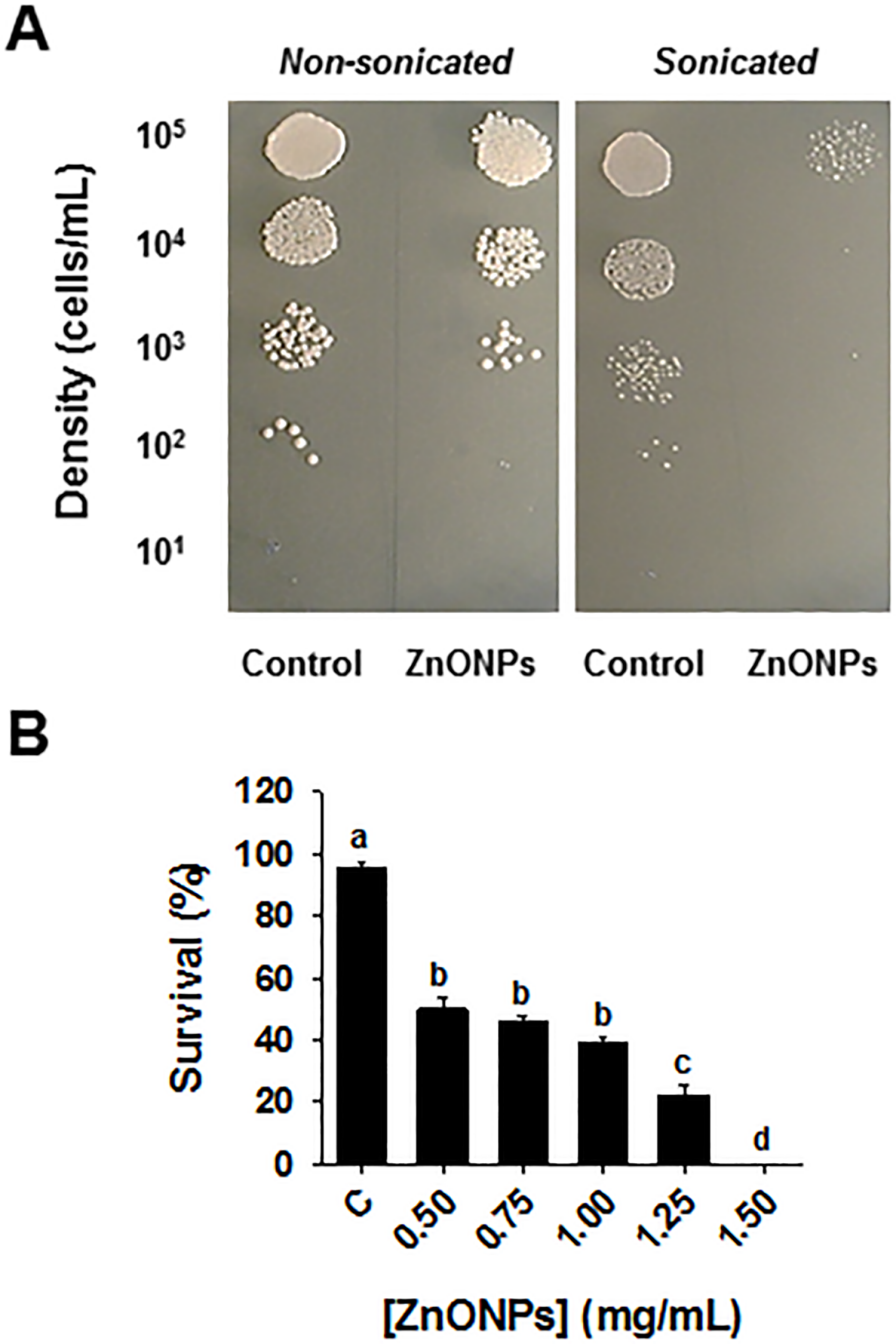
ZnONPs compromise cell wall integrity. (A) Cells were exposed to various concentrations of ZnONPs, subjected to mild sonication, diluted and then spotted onto YPD and compared to non-sonicated counterparts. (B) Cell survival rate (% survival sonicated/non-sonicated cells) was then quantified. Mean ± SEM are presented (n ≥ 3). Significant differences are indicated with letters.

Another major group of mutants that was highly sensitive to ZnONPs had deletions in cell wall organization and biogenesis genes (*p-value ≤* 9.8x10^-3^), and represented 12% of the most sensitive mutant strains (Fig 2A). Included in this group were gene deletions such as *KRE6*, *HOC1* and *BCK1*, involved in glucan biosynthesis, cell wall mannan biosynthesis and control of cell integrity, respectively. Any alteration in cell wall composition caused by gene deletions may modify cell wall rigidity and lead to a higher sensitivity to chemicals that target cell wall integrity. To test the effect of ZnONPs on cell wall functions, a cell wall integrity assay was carried out by exposing ZnONPs-treated cells to mild sonication. The perturbation of the cell wall architecture resulting from a physical agent such as mild sonication can be enhanced by exposing cells to chemicals that interfere with cell wall integrity [55]. Indeed, exposure to ZnONPs enhanced the sonication-induced disruption of yeast cell wall functions in a dose-dependent manner. For example, cells exposed to 0.5 mg/mL ZnONPs displayed a 50% mortality compared to control (non-ZnONPs-treated sonicated cells), whereas a dose of 1.5 mg/mL ZnONPs had no surviving cells (Fig 4). These results provide evidence of an effect of ZnONPs on cell wall sensitivity to sonication.

This experiment supported our inference from the GDA analysis that ZnONPs interfere with cell wall functions in yeast. This effect of ZnONPs on the yeast cell wall resemble the findings by Hassan et al. [56], based on scanning electron microscopy with *Aspergillus* spp., demonstrating that ZnONPs electrostatically interact with cell wall biomolecules to alter the spatial configuration of the cell wall.

### 3.6. Transcription rate analysis in AgNPs-treated yeast

**Fig 5:**
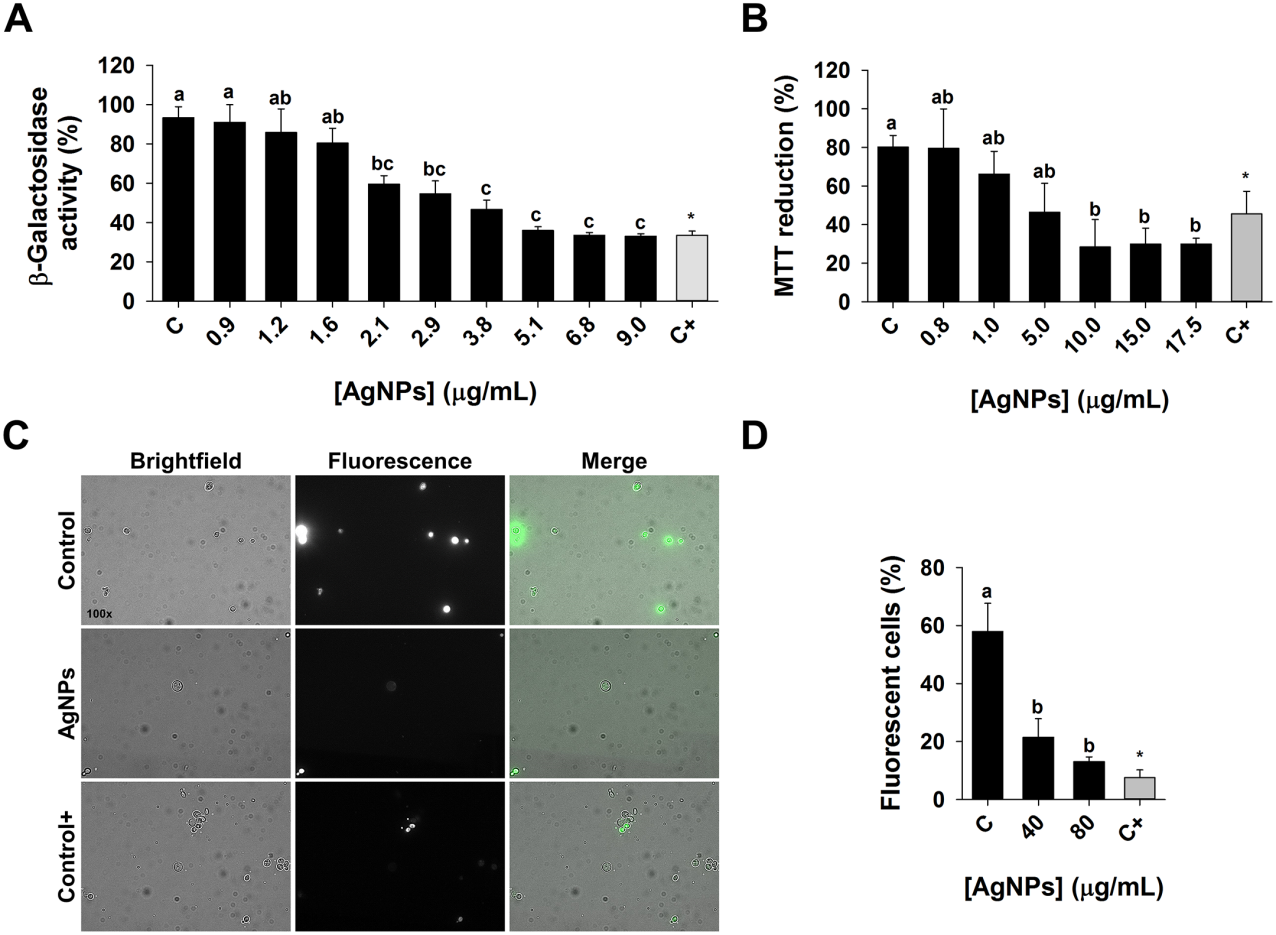
Transcription rate, cellular respiration, and endocytosis in yeast cells exposed to AgNPs. (A) β-galactosidase reporter gene expression assay was used to estimate transcription rate in response to AgNPs or 6-azauracil (positive control). β-galactosidase activity in treatments is expressed relative to no-AgNPs control (negative control). (B) MTT reduction assay was used to estimate cellular respiration in response to AgNPs or sodium azide (positive control). (C) Brightfield, fluorescence, and merged images of negative control, AgNPs (80 μg/mL), and positive control (NaN_3_) groups. The cells that internalized Lucifer Yellow (LY) are fluorescent. (D) The uptake of LY was used to estimate endocytosis in response to AgNPs. Percentage of fluorescent cells relative to control was determined by examining at least 6 different fields, each with >20 cells. Mean ± SEM are presented (n ≥ 3). Significant differences are indicated with letters.

Based on GDA analyses, 16% of the most sensitive deletion mutants to AgNPs were in the transcription and RNA processing category (*p-value ≤* 1.3x10^-3^). Deleted genes included in this group are *THP2* and *THO2* that code for transcription elongation factors, and *CTK1* that codes for a RNA processing protein (S2 Table). To test the effect of AgNPs on transcription, a reporter β-galactosidase expression-based assay was carried out. This assay has been used previously to investigate transcription rates in *Salmonella* [57] and in yeast [58, 59]. The effect of AgNPs on the β-galactosidase enzymatic activity was evaluated as an indirect measure of transcription [46]. Enzymatic activity was estimated in terms of the MUB (4-methylumbelliferon fluorescent product) released after MUG was hydrolyzed by expressed β-galactosidase. The assay showed that AgNPs reduced gene expression in a dose-dependent manner (Fig 5A). For example, exposure of 2.14 μg/mL AgNPs resulted in a 36% decrease in β-galactosidase activity compared to the negative control (no-AgNPs), and a concentration of 9 μg/mL caused a 64% decrease in activity, which is comparable to the effect of 48 μg/mL 6-azauracil, a known inhibitor of transcription.

The effect of AgNPs on transcription may be explained by the ‘hard-soft acid base theory (HSAB)’, which states that ‘soft’ acids such as Ag^+^, among other metals, can bind sulfur or phosphorous in ‘soft’ bases, altering protein structure or function [59–61]. Another potential explanation is that silver ions could be indiscriminately incorporated as metal cofactors into enzymes (ion mimicry), interfering with regular metabolic functions [62, 60]. It is our understanding that the effect of AgNPs on transcription has not been previously reported. More studies are required to determine the specific target of AgNPs in the transcription process.

### 3.7. Cellular respiration analysis in AgNPs-treated yeast

The second largest functional group that resulted in high sensitivity to AgNPs in the GDA analysis comprised mutants lacking genes that code for proteins involved in cellular respiration (14%, *p-value ≤* 5.7x10^-4^). Deleted genes included in this group are *IDH1* (encodes the enzyme for the oxidation of isocitrate to alpha-ketoglutarate), *SOD1* (codes for cytosolic copper-zinc superoxide dismutase that detoxifies superoxide), and *ETR1* (codes for a 2-enoyl thioester reductase that is involved in aerobic respiration). We tested the effect of AgNPs on cellular respiration with the MTT assay. MTT monitors electron transport chain performance, since it is reduced by mitochondrial succinate dehydrogenase to the formazan salt that can be measured spectrophotometrically [63, 47]. Sodium azide is an ETC inhibitor that blocks complex IV in the ETC and was used as a positive control. We observed a 42% reduction in formazan formation when the yeast cells were exposed to 5 μg/mL AgNPs, and a 64% reduction when exposed to 10 μg/mL AgNPs (Fig 5B). The results indicate that the MTT reduction by ETC is inhibited by AgNPs.

Of interest, the level of ETC inhibition by AgNPs at concentration of 10 μg/mL is greater than that of our positive control, 2.5 mM sodium azide (Fig 5B). This might be due to the fact that sodium azide inhibits the heme groups of oxidases, including the cytochrome oxidases (complex IV), but it does not affect the reducing potential from other sources, such as oxido-reductases anchored in non-mitochondrial membranes [64, 65]. It was previously speculated that AgNPs inhibition of *E*. *coli* growth is due to the interaction of silver ions with the thiol groups frequently encountered in membrane and antioxidant proteins, including thioredoxin reductase and superoxide dismutase [66]. Alteration of mitochondrial membrane proteins can trigger permeabilization of membranes and depolarization in mitochondria, provoking an impaired electron transfer that results in oxidative stress [60, 67–68].

### 3.8. Fluid-phase endocytosis analysis in AgNPs-treated yeast

In our high-throughput GDA study, the group representing endocytosis and vesicular transport was also highly enriched among AgNPs-sensitive mutants (Fig 2B). This category represented 12% (*p-value ≤* 1.4x10^-5^) of highly sensitive mutant strains comprising deletions of genes involved in clathrin-mediated endocytosis and vesicular transport, such as *ENT3*, *APM4*, *APL1*, *APL2* and *AAC1*. To further study the effect of AgNPs on endocytosis, a fluid-phase endocytosis assay was performed based on Lucifer Yellow (LY) uptake. LY is a highly hydrophilic dye, whose internalization is mediated by endocytosis rather than passive diffusion [49]. The accumulation of LY can be investigated by fluorescence microscopy and it has been used to assess endocytosis performance [69], where defects in endocytosis can be observed as differences in fluorescence localization and intensity [48, 70]. The fluorescence microscopy assay showed a marked difference in the number of cells internalizing the LY between control and AgNPs-exposed cells (Fig 5C). The percentages of LY-stained yeast cells exposed to 40 and 80 μg/mL AgNPs were 37.1% and 22.6%, respectively, comparable to the positive control (2.5 mM NaN_3_), where only 13.1% of yeast cells were LY-stained (Fig 5D). NaN_3_ inhibits ATP hydrolysis which may interfere with the vacuolar pH balance and thus perturb endocytosis [71, 72]. These observations further validate the results of the GDA screening that suggested AgNPs affect endocytosis.

The effect of AgNPs on endocytosis may be explained by the capability of metal ions to generally impair membrane function [73, 74]. Previous studies demonstrated that AgNPs affected cell membrane morphology in *E*. *coli* and *V*. *cholera*, leading to defective transmembrane transport and increased permeability [75]. Similarly, Kim et al. [76] reported that AgNPs altered the membrane dynamics of *Candida albicans*, changing the chemi-osmotic potential and altering lipid peroxidation. It is well established that modification of membrane dynamics can affect vesicular membrane trafficking [77]. These perturbations can be attributed to the capability of metal-nanomaterials like AgNPs to electrostatically interact with negatively charged functional groups such as COO-, SH- or phosphorous. These functional groups are found in proteins and phospholipids, including those in cellular membranes.

## Conclusions

ZnONPs and AgNPs are commonly used ENMs that have been reported to have a broad spectrum of toxic effects against bacteria, fungi, viruses and algae [9, 10, 65]. The present study extends our understanding of antimicrobial activity using yeast as a model organism, and uncovers complex and distinct modes of action of ZnONPs and AgNPs. Our results support the idea that antifungal activity of ZnONPs is primarily driven by disruption of cell membrane-cell wall complex, and associated processes, such as ion homeostasis. On the other hand, AgNPs inhibit yeast growth by reducing rates of transcription, cellular respiration, and endocytosis. This study is among the first to demonstrate the usefulness of large-scale high-throughput genomics screening approach to study the toxicity of ENMs. The speed and ease of use, coupled with relatively simple data analysis makes chemical-genetic analysis using GDA an ideal tool for the identification of cellular targets that are affected by ENMs.

## Supporting information

S1 Table Highly sensitive yeast deletion mutants to ZnONPs. (DOCX)

S2 Table Highly sensitive yeast deletion mutants to AgNPs. (DOCX)
