# Supplementary material for: Zinc oxide and silver nanoparticles toxicity in the baker's yeast, Saccharomyces cerevisiae
Source: PLoS One. 2018 Mar 19;13(3):e0193111. doi: 10.1371/journal.pone.0193111 (PMC5858749; doi:10.1371/journal.pone.0193111)
Supplement: S1 Table — (DOCX) [file pone.0193111.s001.docx]

### Supplementary Table S1: Highly sensitive yeast deletion mutants to ZnONPs.

| **Gene name** | **Function/Description** | **% Colony size reduction** |
| --- | --- | --- |
| ***A. Transmembrane and membrane transport*** | |  |
| PKR1 | V-ATPase assembly factor, functions with other V-ATPase assembly factors in the ER | 80.70 |
| FEN2 | Plasma membrane H+-pantothenate symporter | 79.30 |
| GUP1 | Plasma membrane protein involved in remodeling GPI anchors | 81.20 |
| ERG2 | C-8 sterol isomerase, catalyzes isomerization at an intermediate step in ergosterol biosynthesis | 78.60 |
| BUD18/ERG28 | Endoplasmic reticulum membrane protein, may facilitate protein-protein interactions between the Erg26p dehydrogenase and the Erg27p 3-ketoreductase | 83.40 |
| ARG82 | Inositol polyphosphate multikinase (IPMK), also has diphosphoinositol polyphosphate synthase activity | 85.20 |
| ERD1 | Predicted membrane protein required for the retention of lumenal endoplasmic reticulum proteins | 88.10 |
| YLR386W/VAC14 | Involved in synthesis of phosphatidylinositol 3,5-bisphosphate, in control of trafficking of some proteins to the vacuole lumen | 69.80 |
| YBR246W/RRT2 | Involved in endosomal recycling; forms complex with Rtt10p that functions in retromer-mediated pathway for recycling internalized cell-surface proteins | 86.40 |
| VPS65 | Dubious open reading frame, unlikely to encode a protein; not conserved in closely related *Saccharomyces* species | 77.10 |
| CCZ1 | Protein involved in vacuolar assembly, essential for autophagy and the cytoplasm-to-vacuole pathway | 80.10 |
| ***B. Ion homeostasis and transport*** | |  |
| CCC2 | Cu(+2)-transporting P-type ATPase, required for export of copper from the cytosol into an extracytosolic compartment | 74.20 |
| FTR1 | High affinity iron permease involved in the transport of iron across the plasma membrane | 79.50 |
| GEF1 | Voltage-gated chloride channel localized to the golgi, the endosomal system, and plasma membrane, and involved in cation homeostasis | 84.60 |
| NHA1 | Na+/H+ antiporter involved in sodium and potassium efflux through the plasma membrane | 72.90 |
| SPF1 | P-type ATPase, ion transporter of the ER membrane involved in ER function and Ca2+ homeostasis | 74.20 |
| VPH1 | Subunit a of vacuolar-ATPase V0 domain, one of two isoforms (Vph1p and Stv1p) | 91.50 |
| AGP3 | Low-affinity amino acid permease, may act to supply the cell with amino acids as nitrogen source in nitrogen-poor conditions | 100 |
| YMR166C | Predicted transporter of the mitochondrial inner membrane | 100 |
| FYV5 | Protein involved in regulation of the mating pathway; binds with Matalpha2p to promoters of haploid-specific genes | 77.10 |
| SOD1 | Cytosolic copper-zinc superoxide dismutase | 82.50 |
| ***C. Transcription and RNA processing*** | |  |
| NAM8 | RNA binding protein, component of the U1 snRNP protein | 63.20 |
| MGA2 | ER membrane protein involved in regulation of OLE1 transcription, acts with homolog Spt23p | 72.50 |
| PHO2 | Homeobox transcription factor; regulatory targets include genes involved in phosphate metabolism | 71.90 |
| PHO4 | Basic helix-loop-helix (bHLH) transcription factor of the myc-family; activates transcription cooperatively with Pho2p in response to phosphate limitation | 66.30 |
| POP2 | RNase of the DEDD superfamily, subunit of the Ccr4-Not complex that mediates 3' to 5' mRNA deadenylation | 73.20 |
| DBR1 | RNA lariat debranching enzyme, involved in intron turnover; required for efficient Ty1 transposition | 55.20 |
| ***D. Cell wall organization or biogenesis*** | |  |
| DFG5 | Putative mannosidase, essential glycosylphosphatidylinositol (GPI)-anchored membrane protein required for cell wall biogenesis in bud formation | 90.60 |
| BCK1 | Mitogen-activated protein (MAP) kinase kinase kinase acting in the protein kinase C signaling pathway, which controls cell integrity | 96.90 |
| HOC1 | Alpha-1,6-mannosyltransferase involved in cell wall mannan biosynthesis | 88.20 |
| KRE6 | Type II integral membrane protein required for beta-1,6 glucan biosynthesis | 81.90 |
| ROM2 | GDP/GTP exchange factor (GEF) for Rho1p and Rho2p | 89.50 |
| SLT2 | Serine/threonine MAP kinase; involved in regulating maintenance of cell wall integrity | 94.70 |
| YLL005C/SPO75 | Meiosis-specific protein of unknown function; required for spore wall formation during sporulation | 79.10 |
| ***E. Cell cycle regulation*** | |  |
| PPH3 | Catalytic subunit of protein phosphatase PP4 complex; regulates recovery from the DNA damage checkpoint and also the gene | 63.50 |
| SPC72 | Component of the cytoplasmic Tub4p (gamma-tubulin) complex, binds spindle pole bodies and links them to microtubules | 78.90 |
| PHO81 | Cyclin-dependent kinase (CDK) inhibitor, regulates Pho80p-Pho85p and Pcl7p-Pho85p cyclin-CDK complexes in response to phosphate levels | 85.10 |
| SCP160 | Essential RNA-binding G protein effector of mating response pathway, mainly associated with nuclear envelope and ER | 73.90 |
| URM1 | Ubiquitin-like protein involved in thiolation of cytoplasmic tRNAs; receives sulfur from the E1-like enzyme Uba4p and transfers it to tRNA | 52.30 |
| ***F. DNA recombination/repair*** | |  |
| RNR3 | Minor isoform of the large subunit of ribonucleotide-diphosphate reductase; regulated by DNA replication and DNA damage checkpoint pathways | 80.20 |
| SML1 | Ribonucleotide reductase inhibitor involved in regulating dNTP production; regulated by Mec1p and Rad53p during DNA damage and S phase | 71.20 |
| BUD32 | Protein kinase, component of the EKC/KEOPS complex required for t6A tRNA modification and may have roles in telomere maintenance and transcription | 70.10 |
| MET18 | DNA repair and TFIIH regulator, required for both nucleotide excision repair (NER) and RNA polymerase II (RNAP II) transcription; involved in telomere maintenance | 77.30 |
| YDR433W (KRE22) | Response to DNA damage | 52.50 |
| ***G. Signaling*** | |  |
| YHL023C/NPR3 | ubunit of SEACIT, a subcomplex of the SEA complex that acts as a GTPase-activating protein (GAP) to negatively regulates signaling | 75.50 |
| DUN1 | Cell-cycle checkpoint serine-threonine kinase required for DNA damage-induced transcription of certain target genes | 55.30 |
| RAM1 | Beta subunit of the CAAX farnesyltransferase (FTase) that prenylates the a-factor mating pheromone and Ras proteins | 71.90 |
| CLA4 | Cdc42p-activated signal transducing kinase of the PAK family, along with Ste20p and Skm1p; involved in septin ring assembly, vacuole inheritance, cytokinesis, sterol uptake regulation | 80.40 |
| ***H. Others*** | |  |
| PHO81 | Cyclin-dependent kinase (CDK) inhibitor, regulates Pho80p-Pho85p and Pcl7p-Pho85p cyclin-CDK complexes in response to phosphate levels | 85.10 |
| YPR100W/MPRL51 | Mitochondrial ribosomal protein of the large subunit | 81.20 |
| HOM6 | Homoserine dehydrogenase (L-homoserine:NADP oxidoreductase), enzyme has nucleotide-binding, dimerization and catalytic regions | 62.40 |
| ILV1 | Threonine deaminase, catalyzes the first step in isoleucine biosynthesis; expression is under general amino acid control | 71.30 |
| PRO2 | Gamma-glutamyl phosphate reductase, catalyzes the second step in proline biosynthesis | 61.50 |
| ***I. Unknown function*** | |  |
| MTC7 (YEL033W) | Unknown function | 51.30 |
| YIL014C-A (YIL015C-A) | Unknown function | 72.50 |
| YML020W | Unknown function | 61.60 |
| YNR073C | Unknown function | 62.30 |
| YOR291W | Unknown function | 69.80 |
| YLR412W | Unknown function | 71.60 |
