# Supplementary material for: Zinc oxide and silver nanoparticles toxicity in the baker's yeast, Saccharomyces cerevisiae
Source: PLoS One. 2018 Mar 19;13(3):e0193111. doi: 10.1371/journal.pone.0193111 (PMC5858749; doi:10.1371/journal.pone.0193111)
Supplement: S2 Table — (DOCX) [file pone.0193111.s002.docx]

**Supplementary Table S2: Highly sensitive yeast deletion mutants to AgNPs.**

| **Gene name** | **Function/Description** | **% Colony size reduction** |
| --- | --- | --- |
| ***A.     Transcription and RNA processing*** | |  |
| GAL80 | Transcriptional regulator involved in the repression of GAL genes in the absence of galactose | 76.7 |
| HMO1 | Chromatin associated high mobility group (HMG) family member involved in genome maintenance | 73.5 |
| HOS1 | Class I histone deacetylase (HDAC) family member that deacetylates Smc3p on lysine residues at anaphase onset | 63.5 |
| NUT1 | Component of the RNA polymerase II mediator complex | 58.9 |
| RLR1/THO2 | Subunit of the THO complex, which is required for efficient transcription elongation and involved in transcriptional elongation-associated recombination | 72.7 |
| SPT4 | Protein involved in the regulating Pol I and Pol II transcription, pre-mRNA processing | 70.2 |
| SSN3 | Cyclin-dependent protein kinase, component of RNA polymerase II holoenzyme; involved in phosphorylation of the RNA polymerase II | 60.8 |
| STP2 | Response to signals from the SPS sensor system for external amino acids | 100 |
| SWI4 | DNA binding component of the SBF complex (Swi4p-Swi6p), a transcriptional activator | 100 |
| TEC1 | Transcription factor targeting filamentation genes and Ty1 expression | 73 |
| THP2 | Subunit of the THO complex, which connects transcription elongation and mitotic recombination | 58.2 |
| PTC1/YDL006W | Type 2C protein phosphatase (PP2C); dephosphorylates Hog1p, inactivating osmosensing MAPK cascade; involved in Fus3p activation during pheromone response | 79.2 |
| YJR084W | Protein that forms a complex with Thp3p; may have a role in transcription elongation and/or mRNA splicing | 86.1 |
| CTK1 | Catalytic (alpha) subunit of C-terminal domain kinase I (CTDK-I); phosphorylates both RNA pol II subunit Rpo21p | 73.4 |
| LOS1 | Nuclear pore protein involved in nuclear export of pre-tRNA and in re-export of mature tRNAs | 65.5 |
| ***B.     Cellular respiration and mitochondrion organization*** | |  |
| IDH1 | Subunit of mitochondrial NAD(+)-dependent isocitrate dehydrogenase | 77.2 |
| SOD1 | Cytosolic copper-zinc superoxide dismutase; some mutations are analogous to those that cause ALS | 79.9 |
| ETR1/YBR026C | 2-enoyl thioester reductase, member of the medium chain dehydrogenase/reductase family | 74.5 |
| AAC3/ YBR085W | Mitochondrial inner membrane ADP/ATP translocator, exchanges cytosolic ADP for mitochondrially synthesized ATP | 68.8 |
| GDS1 | Protein required for growth on glycerol, encountered in mitochondria | 65.5 |
| KGD1/YIL125W | Subunit of the mitochondrial alpha-ketoglutarate dehydrogenase complex; catalyzes a key step in the tricarboxylic acid (TCA) cycle | 96.8 |
| AAC1 YMR056C | Mitochondrial inner membrane ADP/ATP translocator, exchanges cytosolic ADP for mitochondrially synthesized ATP; phosphorylated | 70.7 |
| COQ10/YOL008W | Coenzyme Q (ubiquinone) binding protein | 65.7 |
| PDA1 | E1 alpha subunit of the pyruvate dehydrogenase (PDH) complex | 70.4 |
| INA22/YIR024C | non-tagged protein is detected in highly purified mitochondria in high-throughput | 55 |
| PCP1/YGR101W | Mitochondrial serine protease; required for the processing of various mitochondrial proteins and maintenance of mitochondrial DNA | 61.9 |
| AIM17/YHL021C | protein is detected in highly purified mitochondria in high-throughput studies | 78 |
| ILM1/YJR118C | Protein involved in mitochondrial DNA maintenance | 61.9 |
| ***C.     Endocytosis and vesicular transport*** | |  |
| INP52/YNL106C | Polyphosphatidylinositol phosphatase, dephosphorylates a number of phosphatidylinositols (PIs) to PI; involved in endocytosis | 78 |
| SLA1 | endocytosis; found in the nucleus and cell cortex; has 3 SH3 | 58 |
| LAA1/ YJL207C | Golgi apparatus; involved in TGN-endosome transport; physically interacts with AP-1 | 86.6 |
| ENT3 | Protein containing an N-terminal epsin-like domain involved in clathrin recruitment and traffic between the Golgi and endosomes | 56.2 |
| APM4 | Mu2-like subunit of the clathrin associated protein complex (AP-2); involved in vesicle transport | 86 |
| SEM1 | Component of the lid subcomplex of the regulatory subunit of the 26S proteasome; involved in mRNA export | 77.2 |
| UBP3 | Ubiquitin-specific protease involved in transport and osmotic response; co-regulates anterograde and retrograde transport between the ER and Golgi | 100 |
| APL2 | Beta-adaptin, large subunit of the clathrin-associated protein (AP-1) complex; binds clathrin | 78.5 |
| SLM4/YBR077C | Component of the EGO complex, which is involved in the regulation of microautophagy | 91 |
| SPF1/YEL031W | P-type ATPase, ion transporter of the ER membrane involved in ER function and Ca2+ homeostasis | 93.9 |
| APL1 | Beta-adaptin, large subunit of the clathrin associated protein complex (AP-2); involved in vesicle mediated transport | 61.8 |
| ***D.     Translationand protein processing*** | |  |
| UBA3 | Protein that acts together with Ula1p to activate Rub1p before its conjugation to proteins | 94.3 |
| ICP55/YER078C | Mitochondrial aminopeptidase; cleaves the N termini of at least 38 imported proteins after cleavage by the mitochondrial processing peptidase | 66 |
| DDI1 | DNA damage-inducible v-SNARE binding protein with a role in suppression of protein secretion | 78 |
| MCA1/YOR197W | Ca ^2+^-dependent cysteine protease; may cleave specific substrates during the stress response | 56.8 |
| RAM1 | Beta subunit of the CAAX farnesyltransferase (FTase) that prenylates the a-factor mating pheromone and Ras proteins | 74.7 |
| MRPL51/YPR100W | Mitochondrial ribosomal protein of the large subunit | 84.6 |
| MPRL28/YDR462W | Mitochondrial ribosomal protein of the large subunit | 72.9 |
| RPL8B | Ribosomal protein L4 of the large (60S) ribosomal subunit | 94.4 |
| RPL29 | Protein component of the large (60S) ribosomal subunit, has similarity to rat L29 ribosomal protein | 50 |
| GAL83/YER027C | One of three possible beta-subunits of the Snf1 kinase complex; allows nuclear localization of the Snf1 kinase complex | 50 |
| TOS3 | Protein kinase, related to and functionally redundant with Elm1p and Sak1p for the phosphorylation and activation of Snf1p | 76.2 |
| ***E.     Regulation of cell cycle*** | |  |
| CIK1 | Kinesin-associated protein required for both karyogamy and mitotic spindle organization | 52.8 |
| BUB1/YGR188C | Protein kinase involved in the cell cycle checkpoint into anaphase | 50 |
| AOR1 | Component of the SWR1 complex; complex exchanges histone variant H2AZ (Htz1p) for chromatin-bound histone H2A | 43.4 |
| RTS1/YOR014W | B-type regulatory subunit of protein phosphatase 2A (PP2A) | 50 |
| OPY2/YPR075C | Integral membrane protein that acts as a membrane anchor for Ste50p; involved in the signaling branch of the high-osmolarity glycerol (HOG) pathway and as a regulator of the filamentous growth pathway | 71.3 |
| CTF8 | Subunit of a complex with Ctf18p that shares some subunits with Replication Factor C and is required for sister chromatid cohesion | 60.4 |
| BEM2 | Rho GTPase activating protein (RhoGAP) involved in the control of cytoskeleton organization and cellular morphogenesis | 66.3 |
| YPK1 | Receptor-mediated endocytosis and sphingolipid-mediated and cell integrity signaling pathways | 67.5 |
| YPR097W | Protein that contains a Phox homology (PX) domain and binds phosphoinositide | 89.1 |
| ***F.     DNA damage and stress response*** | |  |
| GPX2 | Phospholipid hydroperoxide glutathione peroxidase induced by glucose starvation that protects cells from phospholipid hydroperoxides and nonphospholipid peroxides during oxidative stress | 82.6 |
| BEM1 | Protein containing SH3-domains, involved in establishing cell polarity and morphogenesis | 66.3 |
| TPS1 | Synthase subunit of trehalose-6-phosphate synthase/phosphatase complex | 83.3 |
| IRC21/YMR073C | Protein involved in resistance to carboplatin and cisplatin | 88.9 |
| ECM4/YKR076W | Omega class glutathione transferase | 81.1 |
| MET18 | DNA repair and TFIIH regulator, required for both nucleotide excision repair (NER) and RNA polymerase II (RNAP II) transcription | 78.7 |
| SLX4 | Endonuclease involved in processing DNA during recombination and repair | 70.5 |
| ARP8 | Nuclear actin-related protein involved in chromatin remodeling | 50 |
| VID31/DEF1 | RNAPII degradation factor; forms a complex with Rad26p in chromatin, enables ubiquitination and proteolysis of RNAPII | 78.7 |
| MRC1/YCL061C | S-phase checkpoint protein required for DNA replication | 41.6 |
| DOT1/YDR440W | Nucleosomal histone H3-Lys79 methylase; methylation is required for telomeric silencing | 71.3 |
| ***G.     Metabolic processes*** | | |
| ARO1 | Pentafunctional arom protein, catalyzes steps 2 through 6 in the biosynthesis of chorismate | 98.1 |
| ARO2 | Bifunctional chorismate synthase and flavin reductase | 50 |
| ARO7 | Chorismate mutase, catalyzes the conversion of chorismate to prephenate | 44.8 |
| URA2 | Bifunctional carbamoylphosphate synthetase, biosynthesis of pyrimidines | 72.5 |
| LPD1/YFL018C | Dihydrolipoamide dehydrogenase, the lipoamide dehydrogenase component (E3) of the pyruvate dehydrogenase | 48.8 |
| DIE2 | Dolichyl-phosphoglucose-dependent alpha-1,2 glucosyltransferase of the ER | 57.6 |
| RBK1 | Putative ribokinase | 50.9 |
| LIP5 | Protein involved in biosynthesis of the coenzyme lipoic acid, has similarity to *E. coli* lipoic acid synthase | 47.1 |
| OPI3 | Phospholipid methyltransferase (methylene-fatty-acyl-phospholipid synthase) | 98.9 |
| CHS3 | Chitin synthase III, catalyzes the transfer of N-acetylglucosamine (GlcNAc) to chitin | 72.5 |
| TES1 | Peroxisomal acyl-CoA thioesterase likely to be involved in fatty acid oxidation rather than fatty acid synthesis | 68.8 |
| ***H.     Others*** | | |
| HOC1 | Alpha-1,6-mannosyltransferase involved in cell wall mannan biosynthesis | 54.5 |
| YLH47/YPR125W | Mitochondrial inner membrane protein exposed to the mitochondrial matrix | 76.5 |
| ECM23/SRD2 | Non-essential protein of unconfirmed function; affects pre-rRNA processing | 81.2 |
| SMI1/YGR229C | Protein involved in the regulation of cell wall synthesis | 57.5 |
| PTK2 | Putative serine/threonine protein kinase involved in regulation of ion transport across plasma membrane | 96.5 |
| ECM4/YKR076W | Omega class glutathione transferase | 62.1 |
| TIP1 | Major cell wall mannoprotein with possible lipase activity | 63.8 |
| MOG1 | Conserved nuclear protein that interacts with GTP-Gsp1p, which is a Ran homolog of the Ras GTPase family | 63.0 |
| EIS1/YMR031C | Component of the eisosome that is required for proper eisosome assembly | 80.4 |
| KIN82 | Putative serine/threonine protein kinase implicated in the regulation of phospholipid asymmetry | 79.2 |
| GRX4/YER174C | Hydroperoxide and superoxide-radical responsive glutathione-dependent oxidoreductase | 64.2 |
| ***I.       Unkown process/function*** | |  |
| DSF2/YBR007C | Unknown function | 72.9 |
| YCR100C | Unknown function | 42.9 |
| JIP4/YDR475C | Unknown function | 33.5 |
| YPK9/YOR291W | Unknown function | 83.7 |
